# Draft Genome of a Type 4 Pilus Defective *Myxococcus xanthus* Strain, DZF1

**DOI:** 10.1128/genomeA.00392-13

**Published:** 2013-06-20

**Authors:** Susanne Müller, Jonathan W. Willett, Sarah M. Bahr, Jodie C. Scott, Janet M. Wilson, Cynthia L. Darnell, Hera C. Vlamakis, John R. Kirby

**Affiliations:** Department of Microbiology, University of Iowa, Iowa City, Iowa, USAa; University of Chicago, Department of Biochemistry and Molecular Biology, Chicago, Ilinois, USAb; The Forsyth Institute, Department of Microbiology, Cambridge, Massachusetts, USAc; Division of Select Agents and Toxins, Office of Public Health Preparedness and Response, Centers for Disease Control and Prevention, Atlanta, Georgia, USAd; Harvard Medical School, Department of Microbiology and Immunobiology, Boston, Massachusetts, USAe

## Abstract

*Myxococcus xanthus* is a member of the *Myxococcales* order within the deltaproteobacterial subdivision. Here, we report the whole-genome shotgun sequence of the type IV pilus (T4P) defective strain DZF1, which includes many genes found in strain DZ2 but absent from strain DK1622.

## GENOME ANNOUNCEMENT

*Myxococcus xanthus* is a soil-dwelling deltaproteobacterium with a genome length of >9.2 Mb. The *Myxococcales* were described in the late 19th century for their capacity to produce macroscopic sporangioles or fruiting bodies (1). Organization into fruiting bodies requires extracellular signaling and coordination of two genetically distinct motility systems (2–29). Previously, the sequence of *M. xanthus* strain DK1622 was determined (NC_008095.1) (30). Recently, we sequenced strain DZ2 (31). These two laboratory strains are noted for behavioral differences (2, 32, 33), and DZ2 has a larger genome (31).

*M*. *xanthus* DZF1 is directly descended from the intermediate strain DK101, the progenitor for DK1622, and displays reduced capacity for type IV pilus (T4P)-mediated motility. An earlier study (34) mapped two point mutations in DK101 to *pilQ*, which encodes the T4P secretin (G741S/N762G), accounting for some phenotypic differences between DZ2 or DK1622 and those strains harboring the *pilQ* allele. Because the DZ2 genome contains additional genes relative to DK1622 and because differences in motility have been noted, we sequenced DZF1 to determine if the additional genes were more likely gained by DZ2 or lost from DK1622, relative to the progenitor.

*M*. *xanthus* DZF1 was sequenced at the University of Iowa DNA Core Facility using 454 GS-FLX titanium technology. Chromosomal DNA was prepared as described previously (31) and processed for sequencing following established protocols. The resulting sequence comprises 388,477 reads totaling 249 Mb, representing 27-fold coverage. The genome was assembled *de novo* into 75 contigs using Newbler software version 2.7. The resulting genome is approximately 9.28 Mb, similar to that for DZ2 (31), and is approximately 147 kb larger than the DK1622 genome. The RAST annotation server (35) predicts a total of 7,704 coding sequences (CDS) within the DZF1 genome.

The *M. xanthus* DZF1 sequence reveals a single nucleotide polymorphism (SNP) in the *pilQ* gene, producing a G741S substitution, but lacks the N762G substitution found in DK101 (34). The impact of these SNPs has not been systematically determined but affects the interpretation of several previous studies. Indeed, deletion of *mazF* (encoding RNA interferase as part of a toxin-antitoxin system) is synthetic with the *pilQ* allele in both DZF1 and DK101 to affect cell death (36, 37).

Current analysis is ongoing to determine the role of genes found in DZF1, but not in DK1622, encoding proteins predicted to function in transcription, translation, signal transduction, fatty acid modification, and protein transport. Homologs to these genes are found in DZ2 as well as other myxobacteria, including *Myxococcus fulvus, Stigmatella aurantiaca*, and *Sorangium cellulosum*. The presence of sequences unique to both DZF1 and DZ2, while absent from DK1622, has been verified by PCR. Thus, the differences between DK1622 and both DZ2 and DZF1 are attributable to a loss of DNA from the DK1622 genome, likely following UV mutagenesis of DK101, which led to excision of one large prophage (38, 39) and may have induced additional lesions. We are investigating several unique sequences found in DZF1 and DZ2 for their role in *M. xanthus* biology.

### Nucleotide sequence accession number.

This whole-genome shotgun project has been deposited at DDBJ/EMBL/GenBank under the accession number AOBT00000000. The version described in this paper is the first version.
